# Bmp and Nodal Independently Regulate *lefty1* Expression to Maintain Unilateral Nodal Activity during Left-Right Axis Specification in Zebrafish

**DOI:** 10.1371/journal.pgen.1002289

**Published:** 2011-09-29

**Authors:** Kelly A. Smith, Emily Noël, Ingrid Thurlings, Holger Rehmann, Sonja Chocron, Jeroen Bakkers

**Affiliations:** 1 Hubrecht Institute, Royal Netherlands Academy of Arts and Sciences (KNAW) and University Medical Center Utrecht, Utrecht, The Netherlands; 2 University Medical Center Utrecht, Utrecht, The Netherlands; 3 Interuniversity Cardiology Institute of the Netherlands, Utrecht, The Netherlands; University of Pennsylvania School of Medicine, United States of America

## Abstract

In vertebrates, left-right (LR) axis specification is determined by a ciliated structure in the posterior region of the embryo. Fluid flow in this ciliated structure is responsible for the induction of unilateral left-sided Nodal activity in the lateral plate mesoderm, which in turn regulates organ laterality. Bmp signalling activity has been implied in repressing Nodal expression on the right side, however its mechanism of action has been controversial. In a forward genetic screen for mutations that affect LR patterning, we identified the zebrafish *linkspoot* (*lin*) mutant, characterized by cardiac laterality and mild dorsoventral patterning defects. Mapping of the *lin* mutation revealed an inactivating missense mutation in the *Bmp receptor 1aa* (*bmpr1aa*) gene. Embryos with a mutation in *lin/bmpr1aa* and a novel mutation in its paralogue, *bmpr1ab,* displayed a variety of dorsoventral and LR patterning defects with increasing severity corresponding with a decrease in *bmpr1a* dosage. In Bmpr1a-deficient embryos we observed bilateral expression of the Nodal-related gene, *spaw*, coupled with reduced expression of the Nodal-antagonist *lefty1* in the midline. Using genetic models to induce or repress Bmp activity in combination with Nodal inhibition or activation, we found that Bmp and Nodal regulate *lefty1* expression in the midline independently of each other. Furthermore, we observed that the regulation of *lefty1* by Bmp signalling is required for its observed downregulation of Nodal activity in the LPM providing a novel explanation for this phenomenon. From these results we propose a two-step model in which Bmp regulates LR patterning. Prior to the onset of nodal flow and Nodal activation, Bmp is required to induce *lefty1* expression in the midline. When nodal flow has been established and Nodal activity is apparent, both Nodal and Bmp independently are required for *lefty1* expression to assure unilateral Nodal activation and correct LR patterning.

## Author Summary

Although vertebrates are bilaterally symmetric when observed from the outside, inside the body cavity the organs are positioned asymmetrically with respect to the left and right sides. Cases where all the organs are mirror imaged, known as situs inversus, are not associated with any medical defects. Severe medical problems occur however in infants with a partial organ reversal (situs ambigious or heterotaxia), which arises during embryonic development. Left-right asymmetry in the embryo is established by unilateral expression of Nodal, a member of the Tgf-ß superfamily of secreted growth factors, a role that has been conserved from human to snails. By performing a genetic screen in zebrafish for laterality mutants, we have identified the *linkspoot* mutant, which displayed partial defects in asymmetric left-right positioning of the internal organs. The gene disrupted in the *linkspoot* mutant encodes a receptor for bone morphogenetic proteins (Bmp), another member of the Tgf-ß superfamily of secreted growth factors. Further analysis of Bmp over-expression or knock-down models demonstrate that Bmp signalling is required for unilateral Nodal expression, through the initiation and maintenance of an embryonic midline barrier. Our results demonstrate a novel and important mechanism by which left-right asymmetry in the vertebrate embryo is established and regulated.

## Introduction

In vertebrates the internal organs are positioned asymmetrically along the left-right (LR) axis. For example, in humans, the heart is positioned on the left side, as is the stomach whilst the liver is positioned on the right side. Within organs LR asymmetry also exists. For example, the two lungs appear identical however they are divided into lobes with 3 on the right lung and 2 on the left. Animals with situs inversus totalis (a LR reversal of all organs) have no pathological features [1] however severe medical problems occur in infants with a partial reversal in a subset of organs (situs ambigious or heterotaxia). These heterotaxic phenotypes occur during early embryonic development and can have both genetic as well as environmental causes [2], [3].

A ciliated organ at the posterior end of the embryo is required for LR-axis specification in the embryo. In this LR organ, the node in mouse or Kupffer's vesicle (KV) in zebrafish, cilia rotate and create a directional fluid flow from the right to left side of the embryo. This directional nodal flow induces a unilateral and asymmetric expression of *Nodal* in the left lateral plate mesoderm (LPM) directing organ laterality. Unilateral expression of *Nodal* is essential for correct LR-axis specification, a function that has been highly conserved from human to snails [2], [4], [5]. Although unilateral expression of *Nodal* is highly conserved and essential for LR–axis specification, there is still very little understanding of how this unilateral *Nodal* expression is initiated by nodal flow and maintained in the LPM.

Nodal is a member of the Tgf-ß superfamily of secreted growth factors. Nodal signaling is activated by the interaction of Nodal ligands with the type I and II Activin receptors and the Cripto coreceptor (reviewed by A.F. Schier [6]). Upon Nodal interaction with its receptor, intracellular Smad2 protein is phosphorylated, which after associating with Smad4 protein is translocated to the nucleus to activate transcription of downstream target genes. Extracellular antagonists such as Lefty and Cerberus can inhibit Nodal signalling either by direct interaction with Nodal or by competing with Nodal for binding to the receptor. The activity of Lefty proteins, Lefty1 and Lefty2, is controlled at the level of transcription. In most tissues Lefty expression is dependent on Nodal signalling [6]. During LR-axis formation in mouse embryos *Lefty1* and *Lefty2* have reciprocal expression patterns. While *Lefty1* is expressed strongly in the presumptive floor plate and only weakly in the left LPM, *Lefty2* is expressed strongly in the left LPM and only weakly in the presumptive floorplate [7]. During LR axis formation in zebrafish embryos *lefty1* is expressed in the notochord. Only after LR patterning has been established are *lefty1* and *lefty2* expressed in the left cardiac field [8]. Nodal likely activates its own expression via a positive feedback loop while it also activates expression of its own antagonists *Lefty1* and *Lefty2*. Genetic experiments in mouse demonstrated that Lefty1 is the more important antagonist and is essential for LR-axis formation [9]. It is believed that *Lefty1* expression in the midline prevents Nodal from crossing the midline, blocking activation of Nodal signalling in the right LPM. Indeed loss of *Lefty1* expression caused the ectopic expression of *Nodal* and other left-sided genes in the right LPM and resulted in various laterality defects. It has been suggested that Nodal and Lefty maintain the L/R asymmetry by a self-enhancement and lateral-inhibition (SELI) mechanism [10]. With the SELI model it is possible to explain how a small difference between two separated regions is converted into a robust difference through local activation and long-range inhibition [11].

Bmps have been implicated in LR patterning but data on their precise role has been contradictory [12]–[22]. This is partly due to Bmp ligands acting in opposite fashions, depending on the time and place of action during LR-axis specification [13], [16]. Bmp proteins are members of the Tgf-ß superfamily of growth factors. Extracellular antagonists of Bmp signalling are Noggin, Chordin and Follistatin. Upon interaction with their serine/threonine kinase type I and II Bmp receptors, Bmp ligands induce intracellular phosphorylation of Smad1, 5 or 8 proteins [23]. Mouse embryos deficient for the type I Bmp receptor Bmpr1a/Alk3 or Acvr1/Alk2 fail to form mesoderm, which has hampered the study of their role during LR-axis specification [24]–[26].

In the current work we describe the identification of the *linkspoot (lin)* mutant from a forward genetic screen for laterality mutants. A missense mutation in the *bmpr1aa* gene is responsible for the LR defect of *lin* mutant embryos. Due to a genome duplication event, there is a second gene encoding a Bmpr1a (*bmpr1ab*) in the zebrafish genome. By screening an ENU-mutagenized zebrafish library we identified a nonsense allele in the *bmpr1ab* gene. Genetic analysis reveals that a reduction in Bmpr1a activity results in left isomerism of the viscera, demonstrating an essential and early role in LR-axis specification. Previous genetic data has provided evidence that Bmp signalling is required to repress Nodal activation in the right LPM but various direct and indirect models have been proposed to explain this activity [12]–[22], [27]. Here we provide evidence that Bmp signalling via Bmpr1a inhibits Nodal activation in the right LPM indirectly by inducing *lefty1* expression in the midline, offering a new model of the interactions between Nodal, Bmp and Lefty in induction and maintenance of LR asymmetry.

## Results

### Identification of the laterality mutant, *linkspoot*, in a forward genetic screen

**Figure 1:**
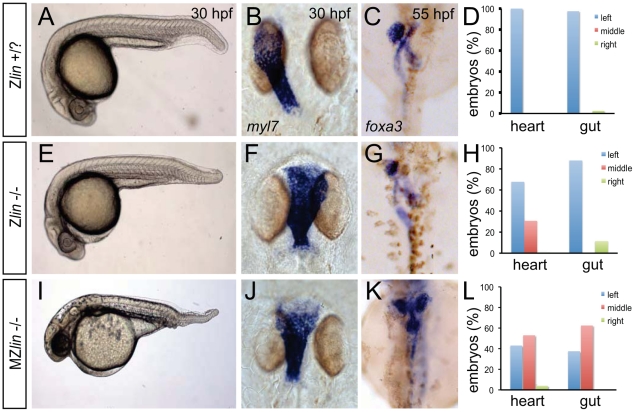
Dorsoventral and laterality defects in zygotic and maternal zygotic *lin* mutant embryos. (A–D) Wild-type zygotic *lin* siblings with normal ventral tail fin (A), left-positioned heart tube (B) and normal organ situs with liver on the left, pancreas on the right and left looped gut tube (C). (D) Quantification of heart position (n = 14) and direction of gut looping (n = 40). (E–H) Zygotic *lin* (*Zlin*) mutant embryos displayed a mild reduction of the ventral tail fin (n = 100/108) (E). In addition, in almost 30% of Z*lin* mutant embryos, the heart tube was positioned at the midline (F). Gut laterality was unaffected in Zlin mutant embryos (G). Quantification of heart position (n = 108) and direction of gut looping (n = 100). (I–L) Maternal zygotic *lin* (*MZlin)* mutant embryos derived from a cross of a homozygous *lin* mutant female and male showing the more severe posterior truncation (I) compared to a Z*lin* mutant embryo (E). In addition, most MZ*lin* mutant embryos displayed a laterality defect in the heart (J), liver (bilateral, K) and in looping of the gut (K). (L) Quantification of heart positioning (n = 151) and direction of gut looping (n = 16).

From an ENU-mutagenesis screen, we identified a unique mutant, *linkspoot* (*lin^hu4087^*), that displayed a reduced ventral tail fin in combination with a heart-specific laterality defect (Figure 1A, 1B, 1E and 1F). At 30 hours post fertilization (hpf), 24.6% (n = 464) of the embryos derived from an incross of two *lin* heterozygous carriers displayed the small but noticeable reduction of the ventral tail fin (Figure 1A and 1E). Whilst the majority of *lin* mutant embryos with the ventral tail fin reduction had no other obvious morphological defects and survived to adulthood, 29% (33 out of 114 *lin* mutant embryos) showed cardiac defects resulting in cardiac failure and death at around 5 days post fertilization (dpf) (Figure 1F, 1G). Examination of the cardiac defect in *lin* mutant embryos revealed a midline positioning of the heart in contrast to a leftward positioning in wild-type siblings at 28 hpf. Furthermore at 48 hpf, when the heart in wild-type sibling embryos has completed looping toward the right, heart looping in these *lin* mutant embryos was incomplete (n = 6/8) (data not shown). Despite the aberrant heart looping in almost 30% of the *lin* mutant embryos, patterning of the myocardium and endocardium was grossly normal. Expression of *tbx2b* and *has2* in the atrioventricular canal myocardium and endocardium, respectively, was comparable between *lin* mutant and sibling embryos (Figure S1). In addition, *bmp4* expression was still restricted (although slightly expanded) to the venous pole, atrioventricular canal and arterial pole (Figure S1).

We observed that the laterality of the other visceral organs (direction of gut looping, positioning of the liver and pancreas) was unaffected in *lin* mutant embryos (30 out of 34) (Figure 1C, 1D, 1G and 1H). Since *lin* mutant embryos that did not display the cardiac defects described above survived up to adulthood we crossed homozygous *lin* mutant females with heterozygous *lin* carrier males. The resulting maternal and zygotic (MZ) *lin* mutant embryos displayed a reduction of ventral structures such as the tail fin and blood islands (Figure 1I). Such phenotypes have been associated with aberrant dorsoventral patterning of the embryo [28]. In addition, we observed in MZ*lin* mutants, uncoordinated laterality defects in the viscera (Figure 1J–1L). Aberrant positioning of the heart and other viscera can be caused by defects in formation or function of the Kupffer's vesicle, resulting in disrupted LR patterning. We therefore examined cilia rotation in the KV, and found that both in Z*lin* and MZ*lin* mutant embryos with a midline positioning of the heart, cilia rotation in the Kupffer's vesicle was unaffected (Figure S2 and Videos S1, S2, S3), suggesting that the laterality defect was not due to a disruption of Kupffer's vesicle function. Together, these results suggest that the affected gene product in *lin* mutants is required for dorsal-ventral and left-right axis specification.

### *linkspoot* encodes Bmpr1aa

**Figure 2:**
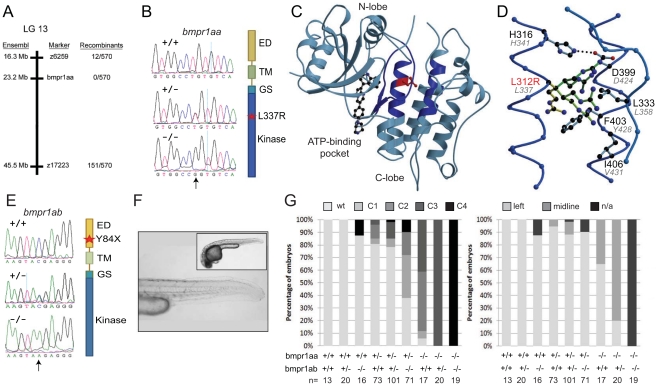
Genetic variations found in zebrafish *bmpr1aa* and *bmpr1ab* genes. (A) The *lin* mutation was mapped to a region on chromosome 13 that includes the *bmpr1aa* gene. (B) T>A basepair change that was found in all *lin* mutant embryos results in a Leu to Arg change at position 337 (L337R). (C) Crystal structure of human BMPR1B. The kinase domain from the human BMPR1B with the kinase inhibitor LDN-193189 (ball-and-stick representation) bound to the ATP binding site (pdb entry 3MDY). Leu 312 (corresponding to Leu 337 in fish) is shown in red. Structural elements providing residues to the hydrophobic core surrounding Leu 312 are highlighted in dark blue. (D) Detailed view of the hydrophobic core surrounding Leu 312 (in red). Black labels refer to the structure of human BMPR1A, the corresponding residues in fish are indicated by grey italic labels. Consequences of the L312R mutation are analyzed by replacing the leucine side chain in the structure model with arginine, of which five typical rotamers are shown (yellow to green). All rotamers cause serious clashes with surrounding residue, which are highly conserved in fish. (E) C>A basepair change in the *bmpr1ab* gene that results in a premature stop codon at position 84 in the extracellular domain of the receptor. (F) A MZ*bmpr1ab* mutant embryo at 2 dpf with no obvious phenotypes in the heart or tail region (magnified). (G) Bmpr1a dose-dependent effect on dorsoventral and left-right patterning. Embryos derived from an incross of *bmpr1aa*+/−;*bmpr1ab*+/− double carrier fish was analyzed and quantified for the dorsoventral phenotypes (classified as C1 (mild) to C4 (strong)) and position of the heart (left or midline) if present. n/a, not applicable since no heart tissue was present.

To better understand the molecular nature of the *lin* mutant phenotype, we positionally cloned the gene that is disrupted in the *lin* mutant. Using bulk segregant analysis with SSLP markers we placed the *lin* mutation onto chromosome 13. Mapping of the *lin* locus using 570 mutant embryos resulted in the identification of a chromosomal region containing a zebrafish orthologue of the mammalian *Bmpr1a/Alk3* gene, encoding a Bmp Type I receptor (Figure 2A). Since Bmp signalling is instructive for cardiac laterality as well as ventral tail fin formation [13], [29], [30], we sequenced the coding region of the *bmpr1aa* gene for mutations. We identified a base pair substitution (T > G) at position 1538 resulting in a leucine to arginine substitution at position 337 (L337R) in the kinase domain of the Bmpr1aa protein (Figure 2B). The T1538G polymorphism was invariably linked with the mutant phenotype (n = 570). No other non-synonymous substitutions were identified in the coding region of *bmpr1aa* that were linked with the mutant phenotype. Modelling of the corresponding region of human BMPR1A suggested that the L312R (corresponding to zebrafish L337R) substitution is incompatible with proper folding of this region and thereby likely destabilizes the entire kinase domain (Figure 2C, 2D).

To address the functional consequence of the L337R substitution, we introduced the *lin* mutation in the *bmpr1aa* gene and generated synthetic mRNA for injection into embryos. Surprisingly, injection of wild-type *bmpr1aa* mRNA into wild-type 1-cell stage embryos resulted in a loss of the ventral tail fin (Table 1). Injection of *bmpr1aa L337R* mRNA had a stronger inhibition of Bmp signalling since more of the injected embryos displayed a dorsalised phenotype, which was also stronger in its effect (Table 1). These results suggest that increasing wild-type Bmpr1a beyond physiological levels has a negative effect on Bmp signalling, possibly by titrating out other components of the signalling pathway. The dominant-negative effect is stronger for the Bmpr1aa L337R most likely because Bmpr1aa L337R is still able to form a receptor complex and interact with Bmp but it can no longer phosphorylate the receptor Smad protein due to its mutation in the kinase domain. To test this hypothesis we injected a lower dose of the wild-type *bmpr1aa* mRNA into embryos derived from an incross of two *lin* heterozygous carriers to determine whether we could rescue the tail fin defects of *lin* mutant embryos. Indeed we observed that injection of low levels of wild-type *bmpr1aa* was able to rescue the ventral tail fin defects in almost 50% of *lin* mutant embryos (Table 1). Consistent with our model that Bmpr1aa L337R has reduced signalling activity, we never observed a rescue of the tail fin defects of *lin* mutant embryos when we injected the *bmpr1aa L337R* mRNA. From these results we conclude that the gene that is disrupted in *lin* mutants encodes the Bmp receptor, Bmpr1aa, and that the *lin* mutation inactivates Bmpr1aa activity.

**Table 1:** Injection studies.

				phenotype#					
RNA injection	conc. ng/µl	genotype	n	wt (%)	C1(%)	C2(%)	C3(%)	C4(%)	†(%)
*bmpr1a*	10	+/+	64	83	3	8	2	0	4
*bmpr1a*	20	+/+	54	72	9	13	0	0	6
*bmpr1a L337R*	10	+/+	70	46	4	14	7	10	19
*bmpr1a L337R*	20	+/+	62	47	10	3	1	23	16
*—*		*lin* +/+,+/−	84	100	0	0	0	0	0
		*lin* −/−	27	11	89	0	0	0	0
*bmpr1a a*	2	*lin* +/+,+/−	81	96	4	0	0	0	0
		*lin* −/−	32	47	53	0	0	0	0
*bmpr1a L337R*	2	*lin* +/+,+/−	78	90	10	0	0	0	0
		*lin* −/−	21	0	100	0	0	0	0

# Classified by strength of dorsalization according to [28].

### Bmpr1aa and Bmpr1ab are partially redundant during dorsal-ventral and left-right axis formation

To further characterise the requirement for *bmpr1a* during zebrafish development, we analysed its expression. Interestingly, database searches revealed that due to a genome duplication event, a paralogue of *bmpr1aa* existed in the form of *bmpr1ab/alk3b* (exhibiting 80% identity at the protein level). We, therefore, simultaneously analysed the expression pattern of these two closely related genes. ISH analysis revealed that both *bmpr1a* paralogues are expressed from the 2-cell stage, indicating maternal deposition of the transcripts (Figure S3). Each paralogue was expressed in a ubiquitous fashion up until the 10-somite stage, however the signal for *bmpr1aa* was more intense compared to the signal of *bmpr1ab* suggesting different levels of expression. From 20-somites onwards, the expression of both paralogues became progressively restricted to anterior regions.

The similar expression patterns observed for *bmpr1aa* and *bmpr1ab* suggest comparable functions for the paralogues. To analyse this possibility further we screened a mutagenesis library for a *bmpr1ab* mutant. We identified a mutant harbouring a stop codon (TAC>TAA) in the second exon of the gene, truncating the protein 84 amino acids into the ligand-binding domain (Y84X) (Figure 2E). Although a DV patterning defect was reported upon morpholino knockdown of *bmpr1ab* [31], we observed no morphological phenotype in the majority of *bmpr1ab* zygotic mutants. Furthermore, maternal zygotic *bmpr1ab* mutants exhibited no observable phenotype (Figure 2F).

We next tested for possible redundancy between the two paralogues. By incrossing double heterozygous carriers for the two mutations (*bmpr1aa*+/−;*bmpr1ab*+/−), we observed a spectrum of dorsalised embryonic phenotypes, ranging from wild-type phenotypes to C4 dorsalisation in the most severe instances (categorisation according to Mullins et al., [28]) (Figure 2G). Genotyping revealed that the severity of the dorsalisation phenotype correlates with decreasing gene dosage of *bmpr1aa* and *bmpr1ab*, with double mutant embryos always exhibiting a C4 dorsalised phenotype. Importantly, this gene dosage effect was also observed on LR patterning, with 80% of embryos of genotype *bmpr1aa*−/−;*bmpr1ab*+/− presenting with a cardiac laterality defect (Figure 2H). Interestingly, loss of the *bmpr1aa* paralogue affected phenotypic severity more robustly than loss of the *bmpr1ab*. Unfortunately, we were unable to score the cardiac laterality phenotype of double mutant embryos as no cardiac field was detected in these embryos (Figure S4), consistent with previous observations that Bmp signalling is required for cardiac specification [32], [33]). These results demonstrate that the *bmpr1a* paralogues play partially redundant roles in both dorsoventral and LR patterning.

### Bmp acts upstream of Nodal signalling during left-right patterning

**Figure 3:**
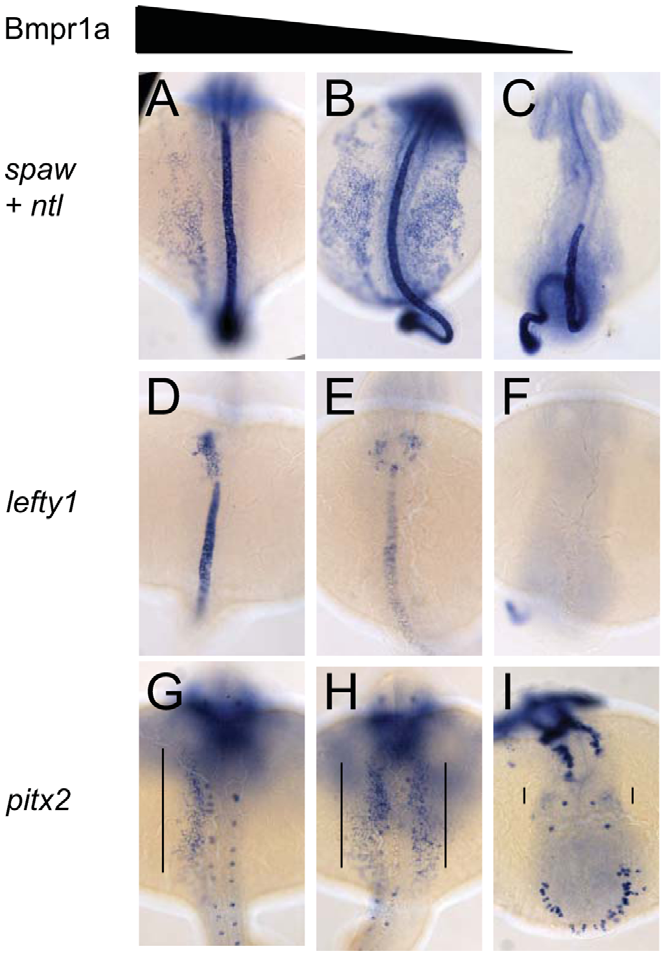
Dose-dependent effect of Bmpr1a on the expression of laterality genes. In situ hybridisation at 18-somites for *spaw* (in LPM) and *no tail* (*ntl*) (in midline) (A–C), *lefty1* at 23-somites (heart field and midline) (D–F) and *pitx2* at 23-somites (in LPM) (G–I). (A,D,G) Embryos selected for normal ventral tail fin or C1 dorsalization (genotypes: *bmpr1aa* +/+ or +/−; *bmpr1ab* +/+ or +/− or −/−). (B,E,H) Embryos selected for C3 dorsalization (genotype *bmpr1aa*−/−;*bmp1ab*+/−). (C,F,I) Embryos selected for C4 dorsalization (genotype *bmpr1aa*−/−;*bmpr1ab*−/−). All embryos are shown as dorsal views with anterior to the top and left to the left. Number of embryos examined is presented in Table 2.

Since no role for Bmp1a in LR axis formation has been reported thus far, we further investigated how Bmp signalling via Bmpr1a regulates LR patterning. We analysed the expression pattern of marker genes whose expression is controlled by LR patterning in embryos derived from an incross of *bmpr1aa*+/−;*bmpr1ab*+/− parental fish. Expression analysis of the Nodal-related gene *spaw* revealed that embryos that retained at least one wild-type copy of *bmpr1aa*, displayed normal *spaw* expression (Figure 3A). However, embryos that had lost both wild-type copy of *bmpr1aa* and retained at least one wild-type copy of *bmpr1ab* displayed strong and bilateral expression of *spaw* in the entire LPM (Figure 3B). On the contrary, in embryos that had lost all wild-type copies of *bmpr1aa* and *bmpr1ab* we observed a reduction of *spaw* expression in the LPM by in situ hybridization (Figure 3C) and quantitative RT-PCR (Figure S5). The *bmpr1aa/bmpr1ab* double mutant embryos displayed a strong (C4) dorsalised phenotype resulting in a curling of the tail region. Although a Kupffer's vesicle was present in these embryos (data not shown), the structure of the tail is suspected to have physically intervened with the potential of the Kupffer's vesicle to activate and/or propagate *spaw* expression in the posterior LPM.

**Table 2:** Expression pattern of *spaw, lefty1*, and *pitx2* in *bmpr1aa/ab* genotypes.

probe	*Bmpr1aa Bmpr1ab*	+/+ +/+	+/++/−	+/+−/−	+/−+/+	+/−+/−	+/−−/−	−/−+/+	−/−+/−	−/−−/−	n
*spaw*	left	1	6	1	3	6	6	3	0	0	26
	bilateral	1	0	0	1	1	0	1	11	0	15
	absent	0	0	0	0	0	0	0	3	15	18
*lefty1*	Left	1	7	3	6	12	2	3	0	0	34
(heart)	bilateral	0	0	0	0	1	0	0	5	0	6
	absent	0	0	0	0	2	1	0	0	4	7
*pitx2*	left	3	7	3	6	7	7	1	0	0	34
(LPM)	bilateral	1	0	0	0	1	2	1	14	0	19
	absent	0	0	0	0	0	0	0	0	3	3

Similar disruptions to asymmetric gene expression were observed upon analysis *of lefty1* expression in the cardiac field and *pitx2* expression in the gut region. Expression of *lefty1* was restricted to the left cardiac field in embryos that retained at least one wild-type copy of *bmpr1aa* (Figure 3D). Embryos that had lost both wild-type copy of *bmpr1aa* and retained at least one wild-type copy of *bmpr1ab*, however, displayed a clear bilateral expression of *lefty1* in the cardiac field (Figure 3E). Since embryos without any wild-type *bmpr1a* gene lack the entire cardiac field, no *lefty1* expression was observed in the cardiac region of these embryos (Figure 3F). Furthermore, *pitx2* is expressed in the posterior LPM and its expression is regulated by Nodal activity; this expression was unaltered in embryos that still possessed at least one wild-type copy of *bmpr1aa* (Figure 3G). Consistent with the observed *spaw* and *lefty1* expression, *pitx2* expression was also bilateral in the LPM of embryos that had lost both wild-type copy of *bmpr1aa* and retained at least one wild-type copy of *bmpr1ab* and was compromised in embryos that had lost all 4 copies of the wild-type *bmpr1a* gene (Figure 3H, 3I).

**Figure 4:**
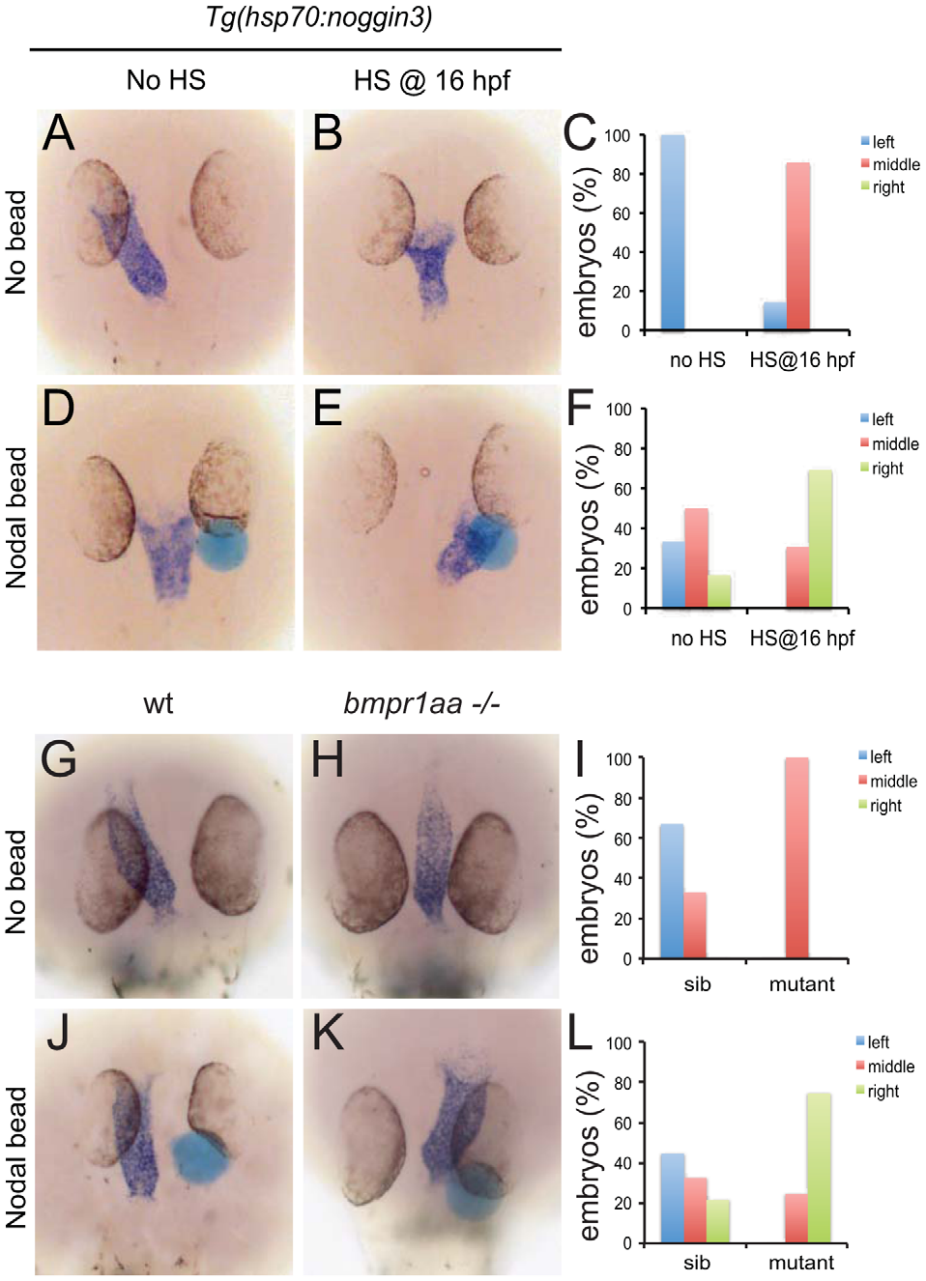
Rescue of Bmp-related cardiac laterality defects by Nodal beads. In situ hybridisation for *myl7* to highlight the position of the linear heart tube at 30 hpf. *Tg(hsp70l:nog3)* embryos with no heat-shock (A,D) or heat-shocked at 16 hpf (B,E). Beads (blue) preincubated with recombinant Nodal protein placed in the right ALPM of non-heat-shocked (D) or heat-shocked (E) *Tg(hsp70l:nog3)* embryos at 17–18 hpf. Control siblings (G,J) or *MZbmpr1aa* mutant embryos (H,K). Beads (blue) preincubated with Nodal protein placed in the right ALPM of siblings (J) or *MZbmpr1aa* mutant embryos (K). Position of the inflow pole of the linear heart tube was determined for embryos without a Nodal bead (C) and for embryos in which a Nodal bead was placed on the right side (F). Embryos are shown as dorsal views with anterior to the top and left to the left.

These results suggest that during LR patterning Bmp signalling via Bmpr1a regulates Nodal activity. To address the interrelation between Bmp and Nodal signalling we tested the possibility that Nodal acts downstream of Bmp signalling during cardiac laterality. Therefore we attempted to rescue the Bmp-dependent cardiac laterality defect by implanting Nodal-soaked beads in the anterior LPM (ALPM), in order to induce ectopic Nodal signalling. To block Bmp signalling, *Tg(hsp70l:nog3)* embryos were heat-shocked at 16 hpf which resulted in a cardiac laterality defect in almost all embryos (6 out of 7; Figure 4A–4C). Interestingly, when a Nodal bead was placed in the right ALPM of non-heat-shocked embryos the heart tube was displaced from the left side towards the midline in approximately 50% of the embryos (Figure 4C, 4F). This effect of the Nodal bead was even stronger when the bead was placed in heat-shocked *Tg(hsp70l:nog3)* embryos. The cardiac tube in such embryos with reduced Bmp signalling was directed towards the right-sided bead in nearly 70% of cases (Figure 4D–4G). In a similar experiment using MZ*bmpr1aa* mutant embryos we again observed that the cardiac tube was directed towards the Nodal bead in 75% of embryos examined (Figure 4H–4J). Together these results suggest that during generation of cardiac laterality Bmp and Bmpr1a act upstream of, or in parallel with, Nodal.

### Expression of *lefty1* in the midline is regulated by Bmp

**Figure 5:**
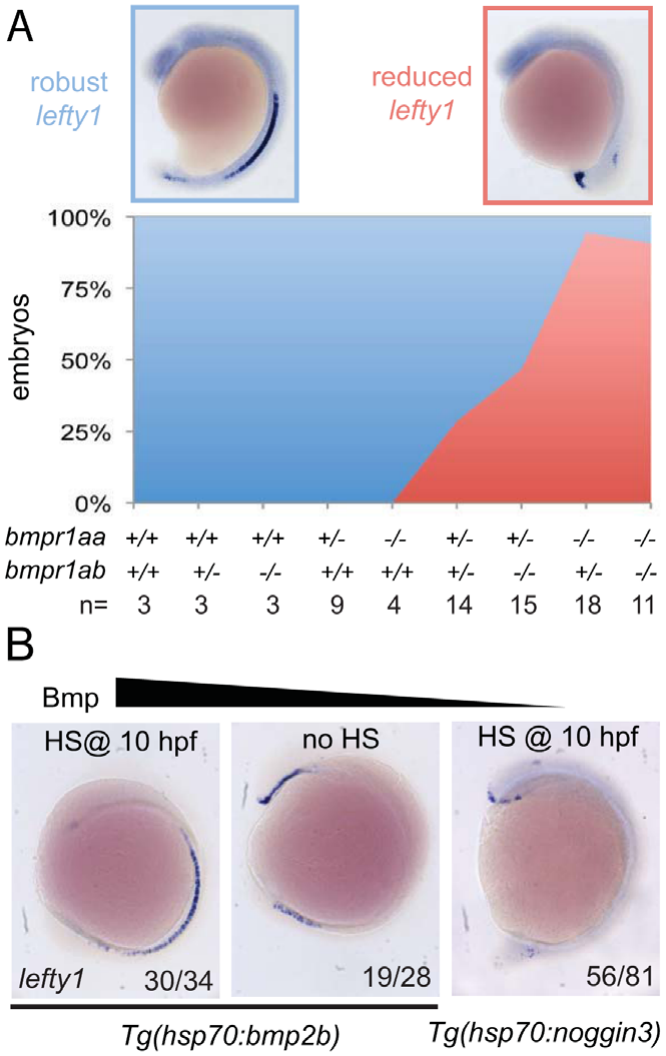
Bmp via Bmpr1a regulates *lefty1* expression in the midline. (A) In situ hybridisation for *lefty1* at 15-somites on embryos from an incross of *bmpr1aa*+/−;*bmpr1ab*+/− double carrier fish. Embryos were analysed for *lefty1* expression and classified as robust (blue boxed panel) or reduced (red boxed panel) expression after which the embryos were genotyped. Quantification of the results is shown in the stacked area graph (blue, robust *lefty1*; red reduced *lefty1*). (B) In situ hybridisation for *lefty1* at 10-somite stage. Embryos shown are *Tg(hsp70l:bmp2b)* embryos either heat-shocked at 10 hpf to induce *bmp2b* expression (left panel) or without heat-shock (middle panel) and *Tg(hsp70l:nog3)* embryos heat-shocked at 10 hpf to inhibit Bmp signalling (right panel). Lateral view of 10-somite stage embryos with dorsal to the right and anterior up.

Our observation that *spaw* is ectopically expressed in the right LPM mesoderm in embryos that had lost 2–3 copies of their wild-type *bmpr1a* indicated that Bmpr1a is normally required to repress *spaw* expression in the right LPM. For this to be a direct effect of Bmp signalling it is expected that Bmp signalling is elevated in the right LPM, as recently reported studying mouse embryos [17]. Although we previously reported on elevated Bmp activity in the left anterior LPM before the cardiac tube is formed (22-somite stage)[34], we never observed enhanced Bmp activity in the right posterior LPM using an anti phospho-Smad1,5,8 antibody (data not shown). An alternative to the model in which Bmp activity directly regulates *spaw* expression in the right LPM is a model in which Bmp activity regulates *spaw* expression in an indirect manner. It is well established that Lefty1 in the midline is required to prevent Nodal protein produced in the left LPM from crossing the midline and inducing *Nodal* expression ectopically in the right LPM [9]. We, therefore, systematically analysed *lefty1* expression in the midline of embryos with a gradual loss of Bmpr1a signalling. Doing so, we observed that embryos with 4 or 3 copies of the wild-type *bmpr1a* gene displayed normal and robust *lefty1* expression in the embryonic midline (Figure 5A). Analysis of *lefty1* expression in embryos that had lost 2 or 3 copies of the wild-type receptor gene, we observed an increase in the number of embryos with reduced *lefty1* expression levels in the midline. Embryos that had lost all 4 copies of the wild-type *bmpr1a* gene consistently showed a near loss of all *lefty1* expression (Figure 5A).

To address whether a disruption of fluid flow in Kupffer's vesicle might explain the reduced *lefty1* expression we analyzed the *lrrc50^hu255h^* mutant, a loss-of-function allele of a conserved cilia protein that is required for cilia motility [35]. We observed that in the majority of *lrrc50^hu255h^* mutant embryos *lefty1* was robustly expressed in the midline (Figure S6), suggesting that the observed reduction of *lefty1* expression in *bmpr1a* mutant embryos was not a consequence of a disruption in Kupffer's vesicle function.

To test whether Bmp activity can regulate *lefty1* expression in the midline we analysed *lefty1* expression in embryos with increased or reduced Bmp activity. We manipulated levels of Bmp signalling by performing heat-shock experiments on embryos carrying the *Tg(hsp70l:bmp2b)* or *Tg(hsp70l:nog3)* transgenes, allowing temporally controlled upregulation or downregulation of Bmp signalling, respectively. The embryos were heat-shocked after gastrulation to prevent strong effects on dorsal-ventral patterning due to altered Bmp signalling levels and *lefty1* expression was analysed at somitogenesis stages. Consistent with the data from the *bmpr1a* mutant analysis, we observed an upregulation of *lefty1* expression in embryos with ectopic Bmp activity, intermediate levels of *lefty1* in wild-type embryos and reduced *lefty1* expression in embryos with reduced Bmp activity (Figure 5B). These results demonstrate that Bmp signalling is both required and sufficient for *lefty1* expression in the midline.

### Bmp and Nodal regulate *lefty1* expression independently

**Figure 6:**
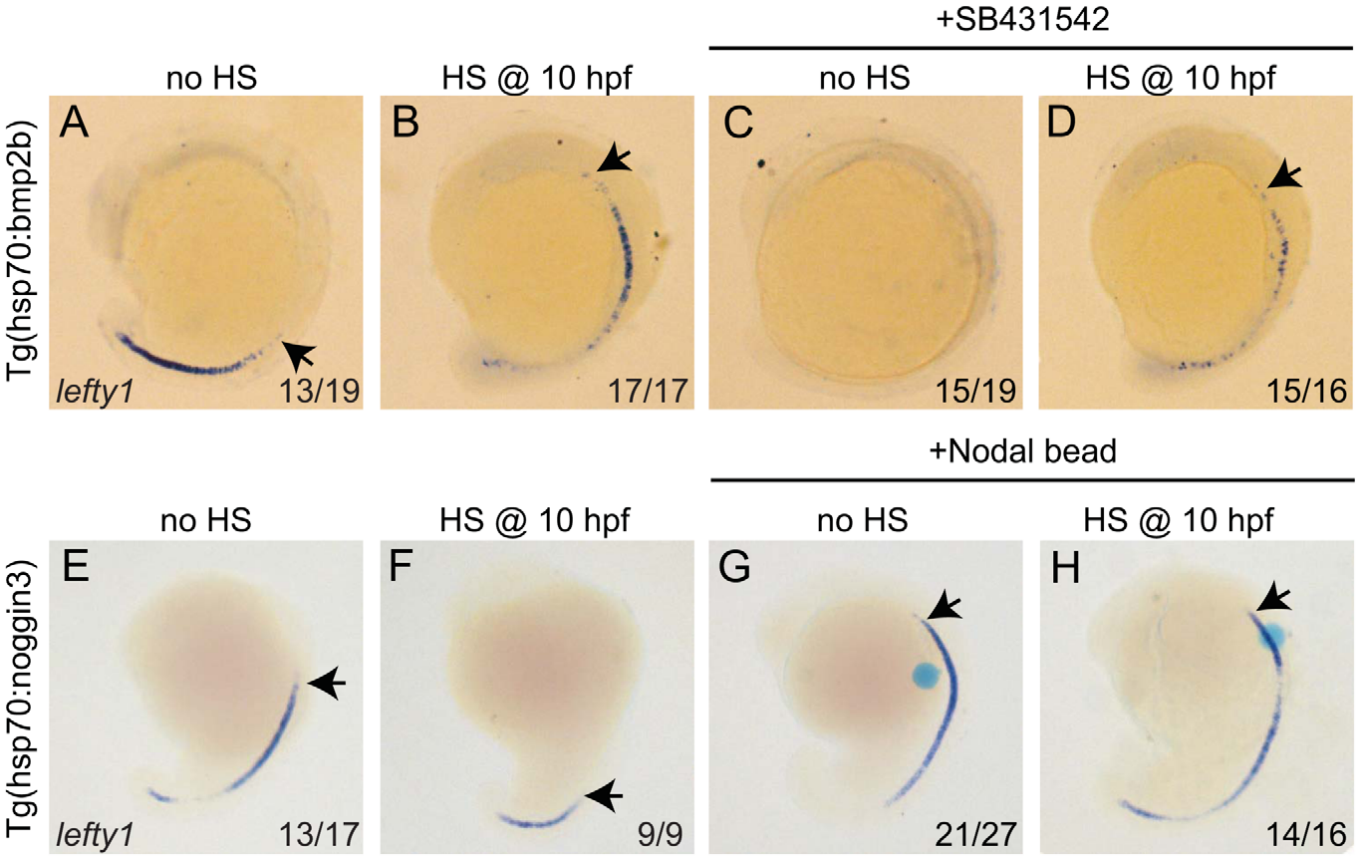
Bmp and Nodal induce *lefty1* independently. (A–D) *Tg(hsp70l:bmp2b)* embryos were left at 28°C (A,C) or heat-shocked at 10 hpf to induce *bmp2b* expression (B,D). A subset of embryos were incubated in the presence of the Nodal inhibitor SB431542 directly after the heat-shock. Embryos were analysed by in situ hybridisation for *lefty1* expression at 15-somites. (E–H) *Tg(hsp70l:nog3)* embryos were left at 28°C (E,G) or heat-shocked at 10 hpf to induce *noggin3* expression (F,H). In a subset of embryos a bead preincubated with recombinant Nodal was placed in the ALPM. Embryos were analysed by in situ hybridisation for *lefty1* expression at 18-somites. All embryos are shown as lateral views with dorsal to the right and anterior to the top. Arrows point to most anterior *lefty1* expression domain. Numbers in lower right represents the number of embryos that displayed the phenotype represented in the panels.

Thus far it has been proposed that *lefty1* expression in the midline is directly regulated by Nodal protein produced in the LPM [36]. Importantly, our observations demonstrate that *lefty1* expression also requires Bmp signalling. Next, we wanted to address whether the regulation of *lefty1* by Bmp is Nodal (in)-dependent. The suggestion that Nodal activity itself is not sufficient to induce *lefty1* expression in the midline arises from our observation that upon reduction of Bmp signalling, *lefty1* expression was reduced while *spaw* was still strongly expressed in the LPM. In addition, our observation that upon ectopic expression of *bmp2b*, *lefty1* expression is induced while *spaw* expression is lost goes further to suggests that Bmp signalling can induce *lefty1* expression in the absence of Nodal activity. We, therefore, wanted to address whether Bmp signalling regulates *lefty1* expression in the midline independent from its regulation by Nodal activity. To investigate further the possibility that Bmp induces *lefty1* expression in a Nodal-independent manner, we incubated embryos with the Nodal inhibitor SB431542 from tail-bud stage until the time point of analysis (18 hpf) [37]. As expected, we observed that in wild-type embryos treated with SB431542, *spaw* expression was compromised in the LPM, indicating the efficiency of the SB431542 treatment in blocking Nodal signalling (data not shown). When wild-type embryos or *Tg(hsp70l:bmp2b)* that were not subjected to heat-shock (both exhibiting wild-type Bmp levels) were treated with SB431542, we observed a loss of *lefty1* expression from the midline. These results demonstrate that Nodal activity is indeed required for *lefty1* expression at this stage, which is consistent with previous reports [5], [38]. However, when heat-shock induced *Tg(hsp70l:bmp2b)* embryos were directly treated with SB431542, *lefty1* expression was induced in the midline. From these results we can conclude that in embryos with wild-type Bmp activity, Nodal is essential to drive robust expression of *lefty1* in the midline. In addition, these results demonstrate that when Bmp signalling is ectopically activated, *lefty1* expression is induced independently of Nodal. When comparing the level of *lefty1* induction in heat-shocked *Tg(hsp70l:bmp2b)* embryos with or without the SB treatment we observed less ectopic *lefty1* expression in the presence of the SB inhibitor (comparing Figure 6B and 6D). This result suggests a synergistic effect of Nodal and Bmp on *lefty1* expression.

Thus far our results suggest that Nodal and Bmp regulate *lefty1* expression in the midline independent from each other. To confirm such an independent regulation we tested whether Nodal can regulate *lefty1* expression independent from Bmp signalling. To block Bmp activity *Tg(hsp70l:nog3)* embryos were heat-shocked at tail-bud stage (10 hpf), which resulted in reduced expression of *lefty1* in the anterior midline at 18 hpf (Figure 6E, 6F). To induce Nodal in Bmp-depleted embryos, a Nodal bead was placed in the ALPM. As a consequence of Nodal bead implantation we observed restoration of the anterior *lefty1* expression even in the absence of Bmp signalling (Figure 6G, 6H). These results demonstrate that Nodal can activate *lefty1* expression independent from Bmp and confirm that Bmp and Nodal regulate *lefty1* expression independent from each other. Together these results indicate that *lefty1* expression is regulated by at least two parallel pathways involving Nodal and Bmp.

### Lefty1 is required for the suppression of *spaw* expression by Bmp

**Figure 7:**
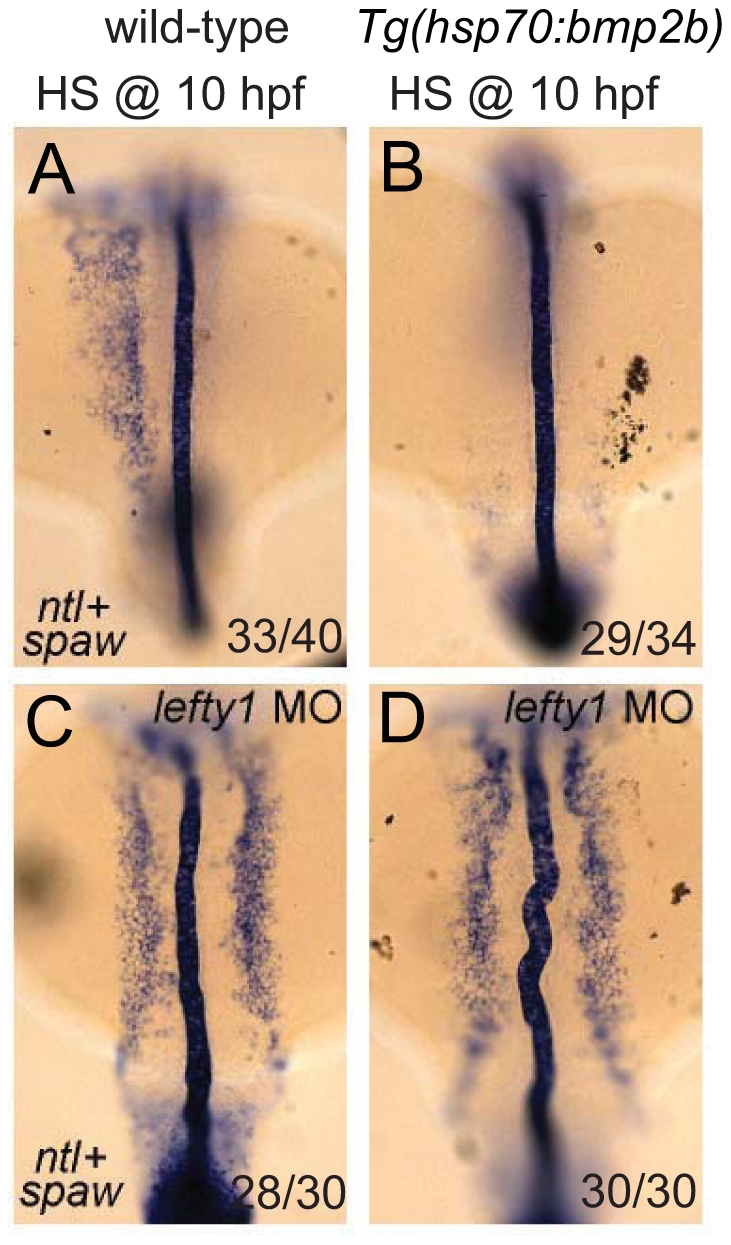
Lefty1 is required for Bmp induced repression of *spaw*. In situ hybridisation of *spaw* (in LPM) and *ntl* (in midline) at 18-somites. Wild-type (A,C) or *Tg(hsp70l:bmp2b)* (B,D) embryos were heat-shocked at 10 hpf to induce *bmp2b* expression (B,D). Ectopic expression of *bmp2b* resulted in the loss of *spaw* expression in the LPM (B). A subset of embryos were injected with a *lefty1* MO (C,D), which resulted in bilateral *spaw* expression even in the presence of ectopic *bmp2b* (D). Embryos are shown as dorsal views with anterior to the top and left to the left.

Finally, we wanted to address whether the observed effect of Bmp on *spaw* expression in the LPM is direct or indirect via its proposed role in regulating *lefty1* expression. In *Tg(hsp70l:bmp2b)* embryos that were heat-shocked at the tail bud stage, we observed a strong down-regulation of *spaw* expression in the LPM (Figure 7A, 7B), which was coupled with ectopic *lefty1* expression (Figure 5B). To test whether the upregulation of *lefty1* in the midline was responsible for the downregulation of *spaw* expression in the LPM, we performed *lefty1* knock-down by injecting embryos with a previously published morpholino that effectively targets *lefty1* [39]. Interestingly, injection of the *lefty1* MO in heat-shock induced *Tg(hsp70l:bmp2b)* embryos resulted in restoration of *spaw* expression in the left LPM, with ectopic expression also observed in the right LPM (Figure 7D), similar to non-heat-shocked embryos (Figure 7C). These results demonstrate that *lefty1* expression in the midline is required for Bmp to repress *spaw* expression in the LPM and acts as an intermediary between Bmp signalling and *spaw* expression.

## Discussion

**Figure 8:**
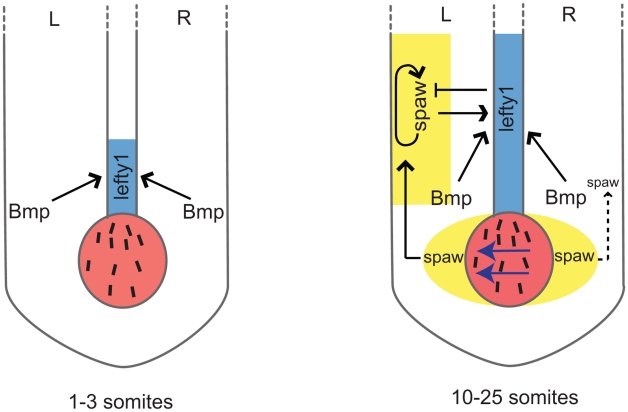
Schematic representation of *lefty1* regulation during LR axis specification. Two phases of *lefty1* regulation can be distinguished. i) At the 1–3 somite stage the KV (shown in red) is formed but the nodal flow (indicated by blue arrows) has not yet been initiated. While at this stage *lefty1* is already expressed in midline (shown in blue) *spaw* expression is still absent from the embryo. Thus, this early *lefty1* expression is induced independent of Nodal but does depend on Bmp activity. Most likely, robust *lefty1* expression is required prior to the initiation of LR axis specification to prevent ectopic activation of *spaw* in the right LPM later on. ii) At the 5-somite stage *spaw* expression becomes apparent in the perinode region (yellow area flanking the KV) and from the 10-somite stage onward (up to the 25-somite stage) *spaw* is expressed unilateral in the left LPM (yellow-boxed area). Our results demonstrate that at this second phase both Spaw/Nodal and Bmp activity are required independently to maintain *lefty1* expression in the midline. Lefty1 in the midline antagonises Spaw and prevents it from crossing the midline where it would induce its own expression in the right LPM.

We describe here the identification of two novel zebrafish *bmpr1a* mutants; a *bmpr1aa* mutant allele from a forward genetic screen for laterality mutants and a *bmpr1ab* mutant allele by screening a mutagenized library. By generating and analyzing compound heterozygous and double mutant embryos for *bmpr1aa* and *bmpr1ab,* we observed a strong correlation between the number of wild-type *bmpr1a* gene copies being lost and the severity of the LR patterning defects observed. Most strikingly we observed a shift from the normal unilateral expression of the Nodal-related *spaw* gene in the left LPM to a bilateral *spaw* expression in both the left and the right LPM. This shift was accompanied by a reduction in the expression of *lefty1* at the midline. This demonstrates that Bmp signalling regulates normal unilateral Nodal activation in the LPM, an observation supported by Nodal bead implantation in the LPM that restored cardiac laterality in Bmp-deficient embryos. Mechanistically our data suggests that there are two parallel pathways, a Bmp and a Nodal dependent pathway, to promote *lefty1* expression in the midline and regulate LR patterning (see Figure 8 for proposed model). This model also explains the observation made in several animal models that ectopic Bmp signalling downregulates Nodal activation, suggesting that Bmp signalling is required on the right side to repress Nodal activation [13], [20], [22], [38]. Our data now demonstrates that, at least in zebrafish, this regulation of Nodal activity by Bmp is indirect and depends on the activation of *lefty1* expression, as was demonstrated by knock-down of *lefty1* in embryos with elevated Bmp signalling (Figure 7). Expanding the previous reaction-diffusion model of an agonist (Nodal) and antagonist (Lefty1), we can now include an additional level of regulation, in which Bmp induces Lefty1, which is required to establish unilateral Nodal activity in the LPM.

Lefty1 is essential for formation of the LR axis [9]. Loss of *Lefty1* in mouse embryos results in a left-isomerism, whereby left-sided genes become expressed bilaterally. These described effects are very similar to those observed upon reducing Bmpr1a levels or Bmp signalling in the zebrafish embryo shown here. Others have reported that expression of *Lefty1* in the midline is dependent on Nodal activity from the LPM in both zebrafish and mouse embryos [5], [36], [38]. Detailed analysis of the *Lefty1* promoter region by Saijoh and colleagues identified a 1.2 kb upstream region of the *Lefty1* gene that was sufficient to drive its midline expression [40]. In addition, it was reported that although Foxh1 binding sites are present in this upstream promoter region, these were not required to drive *Lefty1* expression in the midline [36]. This suggests that, besides Nodal, additional factors are required for inducing midline *Lefty1* expression. Indeed our data demonstrate that during zebrafish LR axis formation, Bmp signalling is required and sufficient to drive *lefty1* expression in the midline. Firstly, we found that in mutants with reduced copies of the wild-type *bmpr1a* gene, *lefty1* expression is gradually lost from the midline. Secondly, in transgenic embryos that ectopically express *noggin3*, a potent Bmp antagonist, *lefty1* expression is diminished from the midline while Nodal signalling is still active (indicated by bilateral *spaw* expression). Thirdly, ectopic activation of the Bmp signalling pathway using a *Tg(hsp70l:bmp2b)* transgenic results in elevated and ectopic expression of *lefty1* in the midline.

Our experiment using ectopic Bmp signalling in the absence of Nodal activity demonstrated that under conditions where Bmp signalling is sufficiently high, Nodal is not required to induce *lefty1* in the midline. This might be important during early stages of LR axis formation. Based on the following observations, we hypothesize that at the initiation of LR axis formation, *lefty1* expression in the midline is initiated by Bmp signalling independently of Spaw activity. Firstly, in zebrafish embryos *lefty1* expression in the midline was observed at the 1–3 somite stage while *spaw* expression is initiated only at the 5-somite stage in the perinode region and at the 10-somite stage in the LPM ([5] and unpublished observations M. Verhoeven, E. Noël and J. Bakkers). Secondly, this initial *lefty1* expression was unaffected by the injection of MOs that efficiently targeted *spaw* [5]. Thirdly, at these early somite stages expression of Bmp ligands is very strong in the tail bud region [41]. When blocking all Bmp signalling at this early stage in heat-shocked *Tg(hsp70l:nog3)* embryos, *lefty1* expression was indeed not initiated in the midline. Together these results suggest that at the initiation of LR axis formation, *lefty1* expression in the midline is initiated by Bmp while the maintenance of *lefty1* expression in the midline requires both Nodal and Bmp (see model in Figure 8).

### Non-redundant roles for Bmp1a and Acvr1l during LR patterning

Although the zebrafish has been used extensively to identify new regulators by conducting forward genetic screens, there has been very limited success identifying novel mutants displaying LR patterning defects [42], [43]. This might be due to the variability and mixture of the phenotypes that can be observed (situs inversus, situs ambigious or situs solitus) as well as the natural occurrence of these phenotypes in the commonly used wild-type strains. Alternatively, an earlier and essential function of the gene product in embryo development masking any LR defects would hamper the identification of such LR genes. In addition, redundancy with paralogous genes often present in the zebrafish genome can mask the full loss-of-function phenotype. In *lin* mutant embryos, two copies of the wild-type *bmp1aa* gene are lost while the two wild-type *bmpr1ab* copies are still present. The *lin/bmpr1aa* mutant embryos displayed heart-specific laterality defects (although not fully penetrant) without displaying any gut laterality defects. Previously, we showed temporally distinct requirements for Bmp signalling functions during both LR axis formation and heart morphogenesis [13], [34]. The heart-specific laterality defect of *lin/bmpr1aa* mutant embryos (eg. loss of leftward cardiac jogging and rightward cardiac looping) is very similar to the cardiac laterality defect previously observed in the *lost-a-fin/alk8* mutant. This suggests that during these processes Bmpr1a/Alk3 and Acvrl1/Alk8 play non-redundant functions similar to those described for these receptors during dorsoventral patterning [31]. These results also imply that either the regulation of heart laterality is more sensitive to reducing Bmp signalling activity than the digestive system or that this process is less compensated by wild-type maternal *bmpr1aa* RNA present in the oocyte. In agreement with the latter suggestion are the observations that *bmpr1aa* is maternally provided in the oocyte and that maternal zygotic (MZ)*lin/bmpr1aa* mutant embryos (from surviving *lin/bmpr1aa* homozygous females) displayed an increase in the strength of the LR patterning defects, including gut laterality defects.

### Conservation of the Nodal-Bmp-Lefty1 pathway

To our knowledge, this is the first report describing the requirement for Bmpr1a in regulating LR axis formation. Mouse *Bmpr1a* mutant embryos do not form mesoderm at embryonic day 7.5 and subsequently die before embryonic day 9.5, preventing the study of LR axis formation in these mutants [26]. Interestingly, the closely related mouse *Acvr1/Alk2* gene has been implicated in LR patterning [16]. Since the *Acvr1* mutant mouse embryos also die early due to severe gastrulation defects, chimeric embryos were produced and analysed for LR patterning. Depending on the relative contribution of mutant cells to the chimeric embryos, a variety of laterality defects were described. In chimeric embryos with a relative high contribution of *Acvr1* mutant cells, bilateral expression of *Nodal* and *Pitx2* in the LPM was observed in combination with reduced expression of *Lefty1* in the midline. The phenotypes described for the chimeric embryos with *Acvr1* mutant cells corroborate our observations in the *Bmpr1a* compound heterozygous/mutant embryos, suggesting a conserved role for Bmp type I receptors during LR axis formation.

The Bmp signal that regulates *Lefty1* expression in the midline does so independent of Smad1, one of the three Bmp-specific Smad proteins. Although Smad1 inactivation in mouse embryos resulted in the activation of Nodal expression in the right LPM, *Lefty* expression in the midline was unaffected in such embryos [15]. Alternatively, Smad5 could be responsible for transducing the Bmp signal. Embryos lacking Smad5 no longer express *Lefty1* in the midline, which is accompanied by bilateral *Nodal* and *Pitx2* expression in the LPM [12]. Several observations in mouse suggest that during LR axis specification, Bmp signalling can also repress Nodal activation in the right LPM more directly and independently from its regulation of *Lefty1* in the midline. As mentioned above, Smad1-deficient embryos showed bilateral Nodal expression while *Lefty* expression in the midline was reported to be unaffected [15]. In a study by Mine and co-workers, elevated phospho-Smad1,5,8 levels in the right LPM compared with the left LPM of mouse embryos was reported [17]. In addition, an increase on the left side of phospho-Smad1,5,8 levels was observed in *Chordin* and *Noggin* double mutant embryos, combined with a loss of *Nodal* and *Lefty1,2* expression. However in *Chordin;Noggin* double mutant embryos, perinodal *Nodal* was also reduced and defects in the morphology of the node and the density of cilia were described, suggesting an additional defect in the transduction of a signal from the node to the LPM in such embryos. This defect in communication between the node and the LPM most likely also explains why we observed a complete lack of *spaw* expression in the LPM of *bmpr1aa;bmp1ab* double mutant embryos. In zebrafish embryos, we did not observe a stronger phospho-Smad1,5,8 level in the right LPM compared to the left side during LR specification. However, at later stages we did observe the opposite in the anterior LPM where phospho-Smad1,5,8 levels were increased on the left side [34]. In addition, our observation that ectopic Bmp signalling in the *Tg(hsp70l:bmp2b)* embryos can no longer repress *spaw* activation in the LPM when Lefty1 is absent makes it very unlikely that such a direct repression of Bmp signalling on *spaw* expression exists in the zebrafish embryo. Together this indicates that the regulation of *lefty1* by Nodal and Bmp during LR axis specification is conserved amongst various vertebrate species. However there are species-specific differences as to what other activities Bmp signalling has during this process. Possibly, differences in geometry or scale of the embryos and speed of their development might require additional regulatory mechanisms to maintain the crucial but very unstable unilateral Nodal activation during LR axis specification.

## Materials and Methods

### Zebrafish strains and screen

All zebrafish strains were maintained in the Hubrecht Institute using standard husbandry conditions. Animal experiments were approved by the Animal Experimentation Committee (DEC) of the Royal Netherlands Academy of Arts and Sciences. The *bmpr1a* ^hu4087^ mutant was identified during a forward genetic screen performed at the Hubrecht institute. ENU mutagenesis was performed as previously described for the creation of the Hubrecht Institute target selected mutagenesis library [44]. F1 progeny of mutagenised males were outcrossed to create approximately 300 F2 families, which were then incrossed. F3 progeny were screened for cardiac laterality defects at 28–34 hpf. The *bmpr1a* ^hu4087^ mutant can be identified using nested PCR with the following primers:

PCR1

Forward primer: AGCTCATCCGGAGAAGTATG

Reverse primer: TCCACTTCATTTGTGTCACTG

PCR2

Forward primer: TGTAAAACGACGGCCAGT ATATGTACCCAGCCCTGATG

Reverse primer: AGGAAACAGCTATGACCAT AGCTTCAGATTCAGATCAACAC

The *bmpr1ab^sa0028^* mutant was identified from the mutagenesis library at the Sanger institute by screening finclip DNA using nested PCR with the following primers:

PCR1:

Forward primer: CCAGACTACATGCTTCATG

Reverse Primer: ATTGTGACAGGCCTACAATG

PCR2:

Forward primer: TGTAAAACGACGGCCAGT CAGAAGATGCCACAAACAAC

Reverse primer: AGGAAACAGCTATGACCATGGTCACACCGAGTAATTTCC

Products were then sequenced with M13F or M13R primers.

Published transgenic lines used were *Tg(hsp70ll:nog3)^fr14^* and *Tg(hsp70ll:bmp2b)^fr13^* [13].

### Genetic mapping and genotyping

Meiotic mapping of the *linkspoot* mutation was performed using standard simple sequence length polymorphisms. The primers used for SSLP can be found on www.ensembl.org.

### Morpholino oligo and RNA synthesis

The lefty1 morpholino was described previously [39].

The coding region of the *bmpr1aa* gene was cloned into pCS2+ by PCR amplification. The *lin* mutation was introduced in the pCS2+ *bmpr1aa* construct using the QuickChange kit (Stratagene). *In vitro* transcription was performed from Acc65I digested template using the SP6 mMessage mMachine kit for all injected mRNA (Ambion).

### SB431542 treatment

SB431542 (Sigma) was resuspended in DMSO to a concentration of 10 mM, and subsequently diluted to a working concentration of 150 µM in embryo medium. Control embryos were treated with an equal volume of DMSO. 30 embryos were treated per 5 ml of SB/DMSO solution.

### In situ hybridization

In situ hybridization was carried out as previously described [45]. Embryos were cleared in MetOH and mounted in benzylbenzoate/benzylalcohol (2:1) before pictures were taken. Riboprobes were generated by transcription from a linearized template in the presence of 11-UTP.

### Bead implants

Agarose beads (Affigel blue, BioRad) were rinsed twice in PBS and incubated for 1 hr at 37°C with 50 µg/ml recombinant mouse Nodal protein (R&D systems). Implants were performed as previously described [46].

## Supporting Information

Figure S1 Formation of the cardiac atrioventricular canal is unaffected in *bmpr1aa* mutant embryos. (A,B) In situ hybridization for *bmp4* in the heart of wild-type and *bmpr1aa* mutant embryos at 48 hpf. Bmp4 is expressed in the inflow region, atrioventricular (AV) canal (arrow) and outflow region of the heart. Although cardiac looping was affected in *bmpr1aa* mutant embryos, expression of *bmp4* was unaffected. (C,D) In situ hybridization for *tbx2b*, which was expressed in the AV canal in wild-type siblings (C) and *bmpr1aa* mutant embryos (D). (E,F) In situ hybridization for *has2*, which was expressed in the endocardial cushion cells that will form the AV valves. *Has2* expression was unaffected in *bmpr1aa* mutant embryos (F) compared to its wild-type siblings (E).
(PDF)

Figure S2 Cilia rotation in Kupffer's vesicle of Z*lin* mutant is unaffected. Brightfield images of the heart of wt and zygotic *lin* mutants after imaging cilia in the KV. Zygotic *lin* mutants display defects in positioning of the heart, however cilia motility in the KV is unaffected (Videos S1 and S2), demonstrating cilia-independent heart defects.
(PDF)

Figure S3 Expression of *bmpr1aa* and *bmpr1ab*. In situ hybridization for *bmpr1aa* (upper row) and *bmpr1ab* (lower row) at the indicated stages from 2-cells up to 24 hpf. Both maternal *bmpr1aa* mRNA and *bmpr1ab* mRNA was detected at the 2-cell stage. mRNA for both Bmp receptors was detected at the various developmental stages up to 24 hpf. Whilst expression of both Bmp receptors was distributed ubiquitously up to the 10-somite stage, it became progressively more intense in anterior structures at the 20-somite stages and later.
(PDF)

Figure S4 *bmpr1aa/bmpr1ab* double mutant embryos lack myocardial tissue. In situ hybridization for *myl7* (*cmlc2*) expressed in the myocardium of wild-type, *bmpr1aa* mutant or *bmpr1ab* mutant embryos. *Myl7* expression was not detected in *bmpr1aa/bmpr1ab* double mutant embryos. All embryos shown as dorsal views at 30 hpf.
(PDF)

Figure S5 *spaw* expression is affected in *bmpr1aa;bmpr1ab* embryos. RT-PCR analysis of *spaw* expression in wild type – C2, C3 and C4 dorsalised embryos derived from an incross of *bmpr1aa+/-* and *bmpr1ab+/-* heterozygous fish (see 1 for detailed protocol). C3 dorsalised embryos (*bmpr1aa-/-;bmpr1ab+/-*) exhibit a 1.6-fold increase in *spaw* expression, while C4 dorsalised embryos (*bmpr1aa-/-;bmpr1ab-/-*) have a 6.9-fold decrease in *spaw* expression, consistent with in situ analysis of *spaw* expression. *MyoD* expression is gradually reduced in C3 and C4 dorsalised embryos when compared to controls, consistent with a reduction in tail structures.
(PDF)

Figure S6 *lefty1* expression in *lrcc50* mutant embryos. In situ hybridization analysis of *lefty1* expression in *lrrc50* mutant embryos at 16 somites. The majority of wild type embryos express *lefty1* from the posterior tip of the notochord anteriorly to around the middle of the trunk (A). The majority of *lrrc50* mutants express *lefty1* in a similar domain to wild type embryos (B). A subset of *lrrc50* mutants either express *lefty1* in a domain restricted to the posterior tip of the notochord (C), or do not expression *lefty1* (D). Lateral views, dorsal to the right.
(PDF)

Text S1 Supplemental methods.
(DOC)

Video S1 High speed image of cilia rotation in the KV of a wild type embryo at 8 somites. See Text S7.
(AVI)

Video S2 High speed image of cilia rotation in the KV of a zygotic *lin-/-* embryo at 8 somites.
(AVI)

Video S3 High speed image of cilia rotation in the KV of a maternal-zygotic *lin-/-* embryo at 8 somites.
(AVI)
