# Supplementary figures and images for: Bmp and Nodal Independently Regulate lefty1 Expression to Maintain Unilateral Nodal Activity during Left-Right Axis Specification in Zebrafish
Source: PLoS Genet. 2011 Sep 29;7(9):e1002289. doi: 10.1371/journal.pgen.1002289 (PMC3183088; doi:10.1371/journal.pgen.1002289)

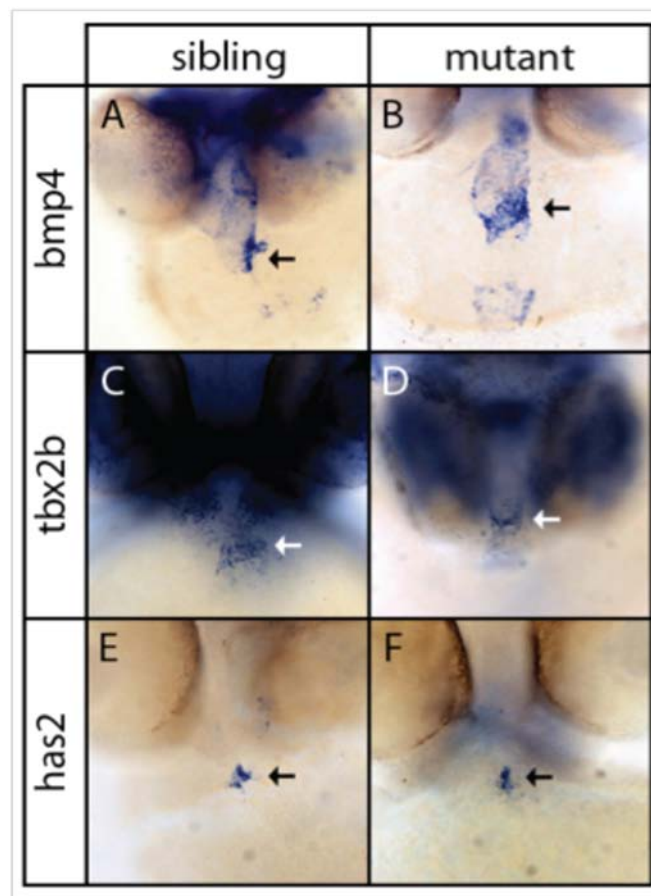

Figure S1

Supplement: Figure S1 — Formation of the cardiac atrioventricular canal is unaffected in bmpr1aa mutant embryos. (A,B) In situ hybridization for bmp4 in the heart of wild-type and bmpr1aa mutant embryos at 48 hpf. Bmp4 is expressed in the inflow region, atrioventricular (AV) canal (arrow) and outflow region of the heart. Although cardiac looping was affected in bmpr1aa mutant embryos, expression of bmp4 was unaffected. (C,D) In situ hybridization for tbx2b, which was expressed in the AV canal in wild-type siblings (C) and bmpr1aa mutant embryos (D). (E,F) In situ hybridization for has2, which was expressed in the endocardial cushion cells that will form the AV valves. Has2 expression was unaffected in bmpr1aa mutant embryos (F) compared to its wild-type siblings (E). (PDF) [file pgen.1002289.s001.pdf]

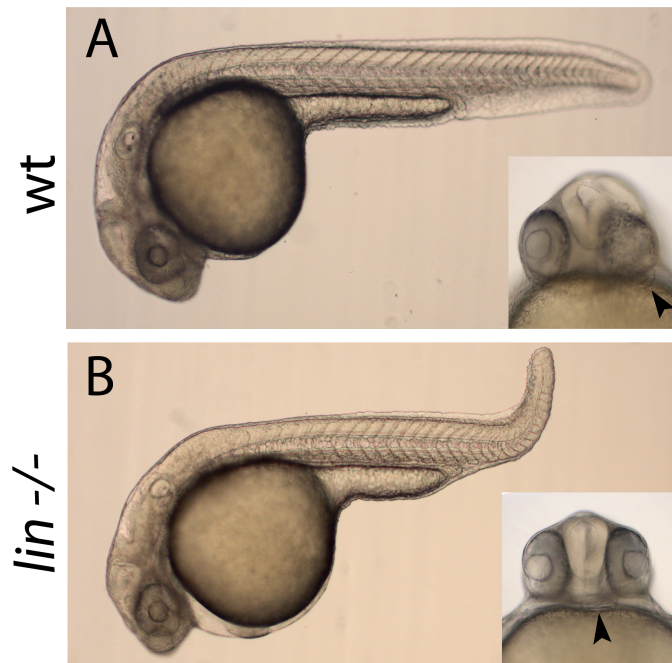

Figure S2

Supplement: Figure S2 — Cilia rotation in Kupffer's vesicle of Zlin mutant is unaffected. Brightfield images of the heart of wt and zygotic lin mutants after imaging cilia in the KV. Zygotic lin mutants display defects in positioning of the heart, however cilia motility in the KV is unaffected (Videos S1 and S2), demonstrating cilia-independent heart defects. (PDF) [file pgen.1002289.s002.pdf]

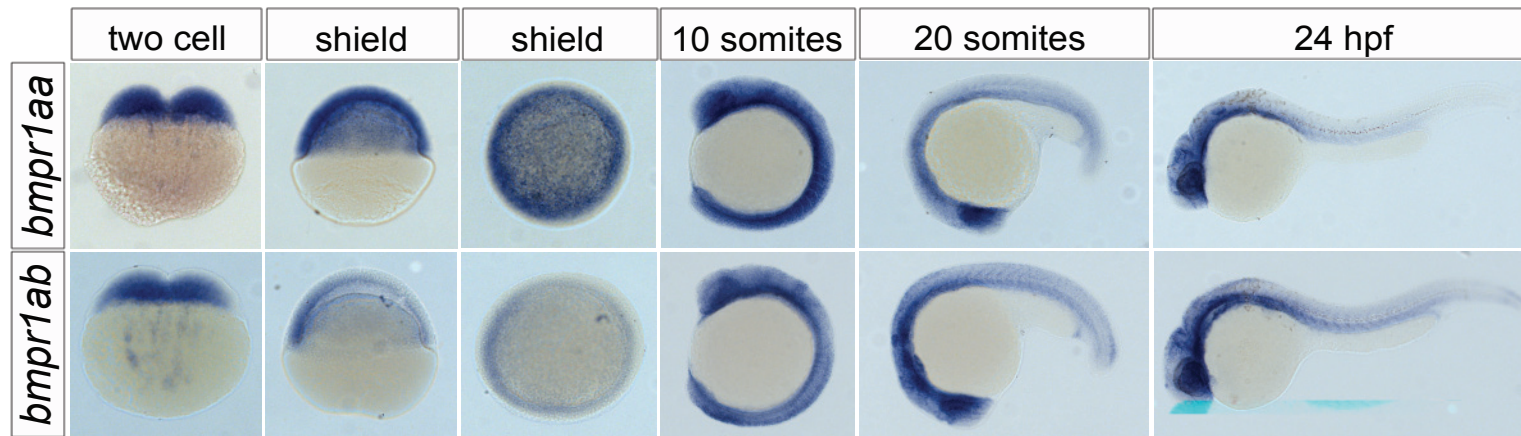

Figure S3

Supplement: Figure S3 — Expression of bmpr1aa and bmpr1ab. In situ hybridization for bmpr1aa (upper row) and bmpr1ab (lower row) at the indicated stages from 2-cells up to 24 hpf. Both maternal bmpr1aa mRNA and bmpr1ab mRNA was detected at the 2-cell stage. mRNA for both Bmp receptors was detected at the various developmental stages up to 24 hpf. Whilst expression of both Bmp receptors was distributed ubiquitously up to the 10-somite stage, it became progressively more intense in anterior structures at the 20-somite stages and later. (PDF) [file pgen.1002289.s003.pdf]

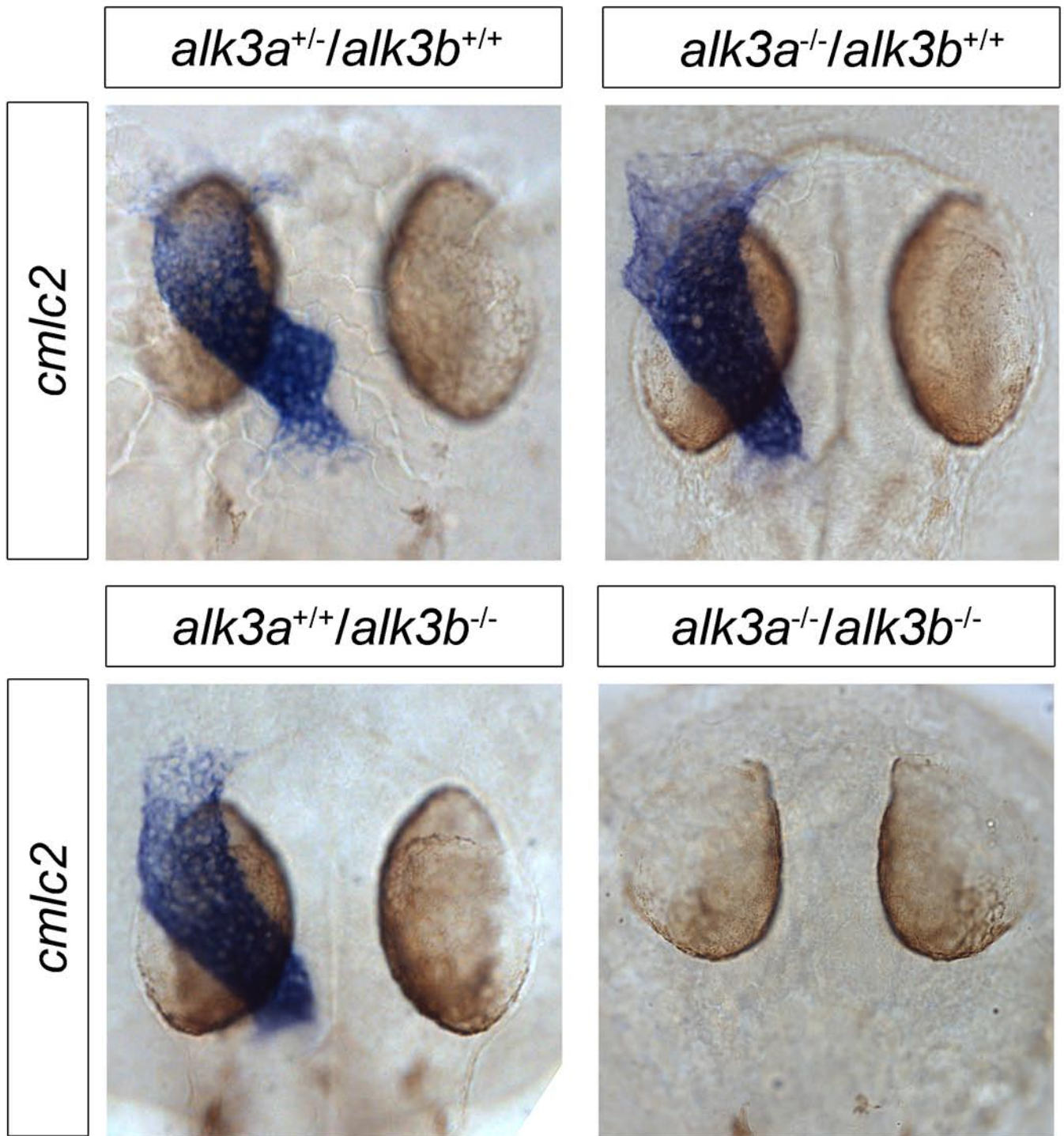

Figure S4

Supplement: Figure S4 — bmpr1aa/bmpr1ab double mutant embryos lack myocardial tissue. In situ hybridization for myl7 (cmlc2) expressed in the myocardium of wild-type, bmpr1aa mutant or bmpr1ab mutant embryos. Myl7 expression was not detected in bmpr1aa/bmpr1ab double mutant embryos. All embryos shown as dorsal views at 30 hpf. (PDF) [file pgen.1002289.s004.pdf]

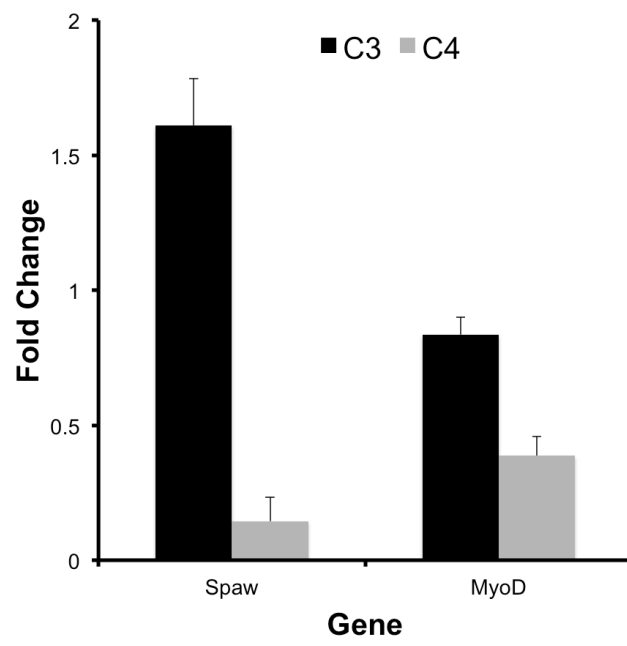

Figure S5

Supplement: Figure S5 — spaw expression is affected in bmpr1aa;bmpr1ab embryos. RT-PCR analysis of spaw expression in wild type – C2, C3 and C4 dorsalised embryos derived from an incross of bmpr1aa+/- and bmpr1ab+/- heterozygous fish (see 1 for detailed protocol). C3 dorsalised embryos (bmpr1aa-/-;bmpr1ab+/-) exhibit a 1.6-fold increase in spaw expression, while C4 dorsalised embryos (bmpr1aa-/-;bmpr1ab-/-) have a 6.9-fold decrease in spaw expression, consistent with in situ analysis of spaw expression. MyoD expression is gradually reduced in C3 and C4 dorsalised embryos when compared to controls, consistent with a reduction in tail structures. (PDF) [file pgen.1002289.s005.pdf]

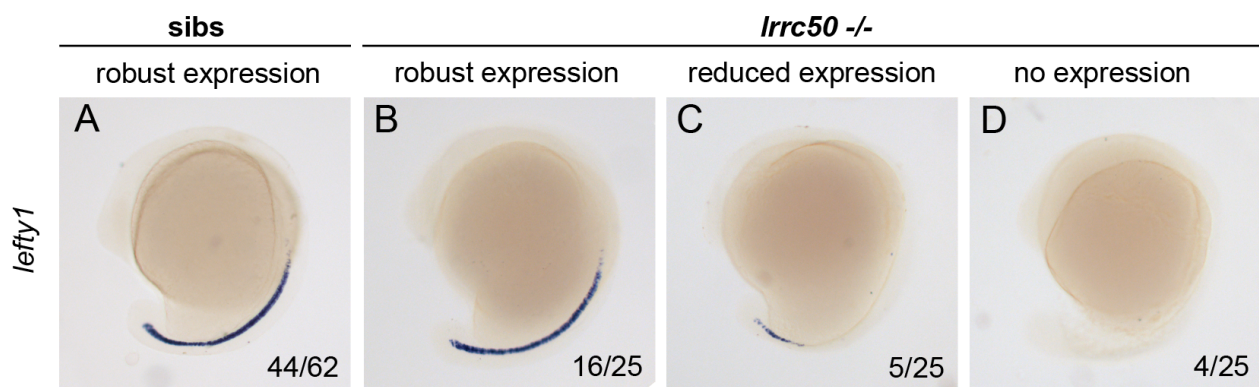

Figure S6

Supplement: Figure S6 — lefty1 expression in lrcc50 mutant embryos. In situ hybridization analysis of lefty1 expression in lrrc50 mutant embryos at 16 somites. The majority of wild type embryos express lefty1 from the posterior tip of the notochord anteriorly to around the middle of the trunk (A). The majority of lrrc50 mutants express lefty1 in a similar domain to wild type embryos (B). A subset of lrrc50 mutants either express lefty1 in a domain restricted to the posterior tip of the notochord (C), or do not expression lefty1 (D). Lateral views, dorsal to the right. (PDF) [file pgen.1002289.s006.pdf]
