# Supplementary material for: Bmp and Nodal Independently Regulate lefty1 Expression to Maintain Unilateral Nodal Activity during Left-Right Axis Specification in Zebrafish
Source: PLoS Genet. 2011 Sep 29;7(9):e1002289. doi: 10.1371/journal.pgen.1002289 (PMC3183088; doi:10.1371/journal.pgen.1002289)
Supplement: Text S1 — Supplemental methods. (DOC) [file pgen.1002289.s007.doc]

**Text S1:** Supplemental methods

**Quantitative RT-PCR**

RNA was extracted from pools of 10-20 whole embryos. cDNA was generated using the First Strand Synthesis System (Invitrogen). Primers were designed against;

*spaw*

F: GATGAGTGGATCGTGCATCCTAAG

R: GGTTGGTAGAGCTTCAACAGACTC

*myoD*

F: CCGTTCTGGAACATTACAGTGGAG

R: CTGTCATAGCTGTTCCGTCTTCTC

*ef1α*

F: GAGTTTGAGGCTGGTATCTCCAAG

R: CTCAGTGGAGTCCATCTTGTTGAC

*gapdh*

F: GGATCTGACAGTCCGTCTTGAGAA

R: CCATTGAAGTCAGTGGACACAACC

qPCRs were performed using SYBR Green super mix (BioRad) on a BioRad MyIQ iCycler, and relative expression levels were determined. Quantifications were performed in triplicate per sample, and each experiment was performed in duplicate.

**Live imaging**

Embryos were mounted in 0.25% agarose at the 5 somite stage. The Kupffer’s vesicle was imaged using a Leica AF7000, in conjunction with a Hamamatsu C9300 high-speed digital camera. All image sequences were acquired using Hokawo software (v2.1) at a rate of approximately 1000 frames per second, for 5 seconds. Post-acquisition, image sequences were slowed to a 250msec frame interval to allow visualization of cilia rotation. Embryos were subsequently removed from agarose, and scored at 28hpf for dorsalisation and cardiac phenotypes.
